# Explainable machine learning model to predict 6-month exclusive breastfeeding: a prospective cohort study in Jiangsu, China

**DOI:** 10.1186/s12884-026-08671-8

**Published:** 2026-01-26

**Authors:** Qian Wu, Chintana Wacharasin, Yan Tang

**Affiliations:** 1 Faculty of Nursing, Jiangsu Medical College, Tinghu District, No. 283, Jiefang South Road, Yancheng, 224005 People’s Republic of China; 2 Faculty of Nursing, Burapha University, Muang District, Chon-Buri, 20131 Thailand

**Keywords:** Predictors, Exclusive breastfeeding, 6 months, Machine learning, XGBoost, SHAP

## Abstract

**Background:**

Exclusive breastfeeding (EBF) during the first 6 months is globally recommended for optimal maternal and child health. Nevertheless, adherence to this recommendation remains suboptimal in China. This study aimed to identify predictors of 6-month EBF among mothers in Jiangsu Province by developing an explainable machine learning (ML) model within a prospective cohort design.

**Methods:**

Between August 2022 and March 2023, postpartum women were recruited through multistage random sampling across hospitals of different levels. Data were collected via structured discharge interviews and three follow-up calls using validated instruments. Least Absolute Shrinkage and Selection Operator (LASSO) regression was applied for feature selection. Four ML algorithms, including Extreme Gradient Boosting (XGBoost), Random Forest, Decision Tree, and Logistic Regression, were compared using tenfold cross-validated area under the receiver operating characteristic curve (AUC) in the training set. The best-performing algorithm was then retrained on the full training set and evaluated in an independent validation set. SHapley Additive exPlanations (SHAP) was applied to enhance interpretability.

**Results:**

A total of 374 mothers completed follow-ups. Fewer than half sustained EBF for 2 months, about one-third for 4 months, and only 12.3% for 6 months. XGBoost showed the highest cross-validated performance (mean AUC = 0.75). After retraining, the XGBoost model achieved an AUC of 0.999 in the full training set and 0.853 in the validation set. SHAP analysis identified the most influential predictors in the following order: breastfeeding (BF) intention, subjective norm, perceived control, BF attitude, BF knowledge, maternal education, and exposure to BF education.

**Conclusions:**

Sustaining EBF for 6 months remains challenging. The XGBoost model, interpreted using SHAP, demonstrated acceptable internal performance. It also yielded exploratory yet informative insights into factors influencing 6-month EBF. These findings generate preliminary evidence that may inform locally relevant EBF support efforts and contribute to the growing body of data-driven EBF studies. External validation is required before considering broader applicability.

## Background

Exclusive breastfeeding (EBF) plays a crucial role in promoting optimal health for both mothers and infants. EBF means feeding infants exclusively with breast milk, without any additional liquids or solid foods, except for syrups, drops, and rehydration solutions. WHO recommends EBF for the first 6 months after birth [1]. EBF for the first 6 months reduces the risk of gastrointestinal infections, respiratory infections, and otitis media, as well as decreases hospitalization rates in infants [2]. Adequate coverage of EBF is estimated to prevent approximately 13% to 15% of deaths in children under 5, especially in low- and middle-income regions [3]. For mothers, 6-month EBF accelerates postpartum weight loss [4], delays menstruation [5], and lowers the risks of type 2 diabetes by 50% [6], ovarian cancer by 27% [7], and breast cancer to approximately 2% [8]. Additionally, EBF offers significant socio-economic benefits by reducing household expenditures on infant formula [9].

Despite these well-documented benefits, the practice of EBF for the initial 6 months remains far from universal. The WHO reports that almost 67% of infants do not receive EBF for the recommended duration, with little improvement observed over the past 2 decades [10]. In Canada, although more than 90% of women start with EBF, the proportion decreases sharply to 35.6% by 6 months [11]. In the U.S., EBF at 6 months increased from 20.5% in 2016 to 26.1% in 2022, indicating some progress, but the rate remained relatively low [12]. This issue is also prevalent in China, where approximately 68% of women fail to maintain EBF for 6 months [13]. The widespread implementation of 6-month EBF continues to be a significant challenge for many countries.

Various determinants have been found to affect EBF duration during the first 6 months postpartum. Through a systematic review [14], the researchers categorized these predictors into 4 main groups: (1) Demographic and Socioeconomic Factors, including maternal age, education, maternity leave, parity, household income, and residence; (2) Biomedical and Clinical Factors, such as pre-pregnancy body mass index (BMI), and delivery mode; (3) Belief and Cognitive Factors, including breastfeeding (BF) knowledge, attitude, subjective norm, perception of insufficient milk (PIM), and perceived control; and (4) Behavioral Intention Factors, involving maternal BF intention, exposure to BF education, and BF experience. However, these findings are mainly based on studies conducted in other countries, and cultural differences limit their direct applicability to EBF outcomes in China [9].

In China, despite a declining birth rate, the large population base and rising consumer spending continue to make it a major player in the global infant formula market [15]. The end of the 1-child policy and the shift to 2- and 3-child policies may introduce diversity and uncertainty in maternal beliefs and practices regarding EBF. Although maternity leave in Jiangsu was extended to 158 days in 2022, inadequate maternity protection for women in informal sectors forces many mothers to return to work early [9]. Traditional beliefs further complicate EBF practices. Families, particularly those with grandparent caregivers, often perceive breast milk as insufficient and resort to supplementing with formula or sugary water, undermining effective EBF [16]. China’s exceptionally high cesarean section rates may present another significant barrier to EBF [17]. Furthermore, most EBF studies in China are cross-sectional and have a time point of less than 6 months, often relying on 24-h or 7-day recall, which may lead to an overestimation of EBF rates. Research on psychological factors like PIM and BF intention is still limited. These factors underscore the complexity and uncertainty surrounding the barriers to 6-month EBF in China, which complicates efforts to improve EBF outcomes.

To better understand and address barriers faced by Chinese mothers in sustaining EBF for the first 6 months, it is essential to identify the influencing factors. The Theory of Planned Behavior (TPB) provides a well-established conceptual framework for understanding EBF behaviors, positing that attitude, subjective norm, and perceived behavioral control influence behavior primarily through behavioral intention [18]. Based on the TPB framework and our systematic review [14], the present study examined related variables influencing EBF in the Chinese context. To capture complex and potentially nonlinear relationships among these variables, a machine learning (ML) based approach was applied, and multiple algorithms were compared. The best-performing model was then interpreted using SHapley Additive exPlanations (SHAP), thereby generating exploratory, context-specific insights into determinants of 6-month EBF and informing the development of locally relevant BF support efforts.

## Methods

### Study subjects

This prospective study was conducted in Jiangsu Province, China (August 2022–March 2023). A multistage random sampling method was used to recruit 385 participants. One city in Jiangsu Province was randomly selected by the lottery method. Within that city, 1 hospital was randomly selected from Level I, Level II, and Level III hospitals by the same method. In the selected hospitals, we invited eligible women admitted to the postpartum wards after delivery to participate, according to pre-specified inclusion and exclusion criteria. Only those who met the eligibility requirements and consented to participate were included.

### Inclusion criteria and exclusion criteria

The inclusion criteria for participants were as follows: 1) healthy adult women aged 18 years or older; 2) women who gave birth either vaginally or by cesarean section; 3) women who gave birth to a healthy singleton at ≥ 37 weeks of gestation; 4) women with no specific dietary restrictions; 5) women whose spouse, mother, and mother-in-law are alive; 6) women who communicated fluently in Mandarin; 7) women who possessed a mobile phone available for study follow-up. The exclusion criteria were: 1) women and infants for whom BF was contraindicated due to health issues; 2) women with mental or emotional incapacity to continue participating in the study.

### Data collection and measures

All variables other than EBF practice were collected through face-to-face interviews on the day of hospital discharge. EBF practice was assessed during 3 postpartum telephone follow-ups. Details of the measurements are provided below.

Demographic Record Form: A self-designed form collected data on maternal age, education, maternity leave duration, parity, household income, place of residence, pre-pregnancy BMI, delivery mode, exposure to BF education, and previous BF experience.

Chinese version of BF Knowledge Questionnaire [19]: This 17-item questionnaire assessed maternal BF knowledge, covering 2 dimensions (BF benefits and skills). Correct answers were scored as 1 point, while incorrect or uncertain responses were scored as 0 points. Total scores ranged from 0 to 17, with higher scores indicating better knowledge. The Cronbach’s alpha was 0.74 [19] and 0.86 [20], reflecting good internal consistency.

Chinese Version of the Breastfeeding Attrition Prediction Tool [21]: This tool comprises attitudinal scale, subjective norm scale, and perceived control scale, which assessed BF attitude, subjective norm, and perceived control, respectively. The instrument includes 44 items on a 5-point (1–5) Likert scale, with higher scores indicating stronger BF beliefs or greater acceptance of BF. The overall Cronbach’s alpha coefficient was 0.88, with subscale values of 0.72, 0.83, and 0.86 for the attitudinal, subjective norm, and perceived control scales, respectively.

Chinese Version of the Perception of Insufficient Milk Questionnaire [22]: This questionnaire evaluated maternal PIM. It includes a global question on milk supply perception (answered “yes” or “no”) and five 5-point (1–5) scale items assessing perceived breast milk deficiency. Higher scores indicate a stronger perception of adequate milk supply. The Cronbach’s alpha coefficient was 0.94.

Chinese Version of the Infant Feeding Intention Scale [23]: This scale measures mothers’ BF intention with 5 items scored from 0 to 4. The total score is derived by averaging items 1 and 2, then adding items 3, 4, and 5. A higher score indicates a greater intention to exclusively breastfeed for 6 months. The Cronbach’s alpha was 0.77, and it demonstrated strong construct validity (goodness-of-fit index = 0.997).

EBF practice was measured as the cumulative number of days the infant was exclusively breastfed from birth to 6 months postpartum. During hospitalization, mothers were trained and began completing a daily EBF record form, which they continued each day after discharge. During the telephone follow-ups at 2, 4, and 6 months, mothers reported their cumulative EBF days based on these daily records. Modest incentives were provided to promote adherence and data completeness. For analytic purposes, cumulative EBF days were converted into corresponding EBF duration categories: 60–75 days was classified as sustaining EBF for 2 months, 76–105 days for 3 months, 106–135 days for 4 months, 136–165 days for 5 months, and 166–180 days for 6 months. For the primary analysis, sustained EBF for 6 months was defined as cumulative EBF of 166–180 days, while all shorter durations were classified as non-6-month EBF.

### Statistical analysis

Statistical analyses were conducted in R version 4.3.0 and Python 3.9.12. Data were summarized as mean ± standard deviation or median (interquartile range) for continuous variables and as counts (percentages) for categorical variables. Group comparisons were performed using chi-square, independent t, or Mann–Whitney U tests as appropriate. Missing values (if < 5%) were imputed using the median for continuous variables and the mode for categorical variables. Hyperparameters were tuned using grid search with cross-validation.

Participants were randomly allocated (7:3) into training and validation sets. Least Absolute Shrinkage and Selection Operator (LASSO) regression with cross-validation was used to select predictors in the training set. A variance inflation factor (VIF) greater than 4 and a tolerance value below 0.20 were considered indicative of multicollinearity among the variables selected by LASSO [24, 25]. Models based on four ML algorithms, including Extreme Gradient Boosting (XGBoost), Random Forest, Decision Tree, and Logistic Regression, were trained using the training set. Predictive performance was compared using tenfold cross-validated area under the receiver operating characteristic curve (AUC). The best-performing model was retrained on the full training set and evaluated on the validation set, with performance assessed by AUC (with confidence intervals), sensitivity, specificity, precision, F1-score, and calibration curves. SHapley Additive exPlanations (SHAP) were used to interpret predictor contributions. Statistical significance was set at *p* < 0.05.

### Ethics approval

The study was approved by the Institutional Review Board of Burapha University (G-HS001/2565) and Pizhou People’s Hospital (20,220,906–01). Since Fuyou Hospital and Xinan Hospital do not have independent ethics committees, their ethical review was delegated to the IRB of Pizhou People’s Hospital. Both hospitals also provided formal administrative permission for data collection. This study was conducted in accordance with the Declaration of Helsinki. It involved minimal-risk, questionnaire-based procedures. All participants were fully informed about the study objectives, procedures, potential risks, and their right to withdraw at any time without consequence. Written informed consent was obtained from all participants. To protect privacy, all data were de-identified prior to analysis, and confidentiality was strictly maintained throughout the study.

## Results

### Basic characteristics of participants

This study ultimately enrolled 385 mothers from Pizhou People’s Hospital (*n* = 180; Level Ⅲ), Fuyou Hospital (*n* = 180; Level Ⅱ), and Xinan Hospital (*n* = 25; Level Ⅰ). 374 mothers completed all 3 follow-ups (response rate, 97.14%). Reasons for withdrawal included infant lactose intolerance (*n* = 1), low birth weight (*n* = 1), maternal overseas relocation for study (*n* = 1), maternal illness requiring hospitalization (*n* = 3), and unreachable by phone (*n* = 5). Among the 374 completers, EBF was maintained by 43.58% of participants for 2 months, 41.44% for 3 months, 35.03% for 4 months, 22.99% for 5 months, and 12.30% for 6 months, illustrating a clear decline over time. Baseline characteristics are summarized in Table 1. No significant differences were observed between the training and validation sets (*P* > 0.05).

**Table 1:** Baseline characteristics in the training and validation sets(*n* = 374)

Variables	Total (*n* = 374)	Test (*n* = 111)	Train (*n* = 263)	*p*
Age, Median (Q1-Q3)	30 (27- 33)	30 (26- 33)	30 (28- 33)	0.097
Pre-pregnancy BMI, Median (Q1-Q3)	22.86(20.58- 25.53)	22.66(20.5- 25.52)	22.95(20.69- 25.53)	0.925
Quantity of child, n (%)				0.188
1	197 (53)	66 (59)	131 (50)	
2	164 (44)	43 (39)	121 (46)	
≥ 3	13 (3)	2 (2)	11 (4)	
Residence, n (%)				0.412
Urban	260 (70)	81 (73)	179 (68)	
Rural	114 (30)	30 (27)	84 (32)	
Average monthly household income (RMB), n (%)				0.49
< 3000	3 (1)	1 (1)	2 (1)	
3000–6000	47 (13)	17 (15)	30 (11)	
6000–10000	104 (28)	26 (23)	78 (30)	
> 10,000	220 (59)	67 (60)	153 (58)	
Education, n (%)				0.835
Junior college or below	284 (76)	83 (75)	201 (76)	
Bachelor degree or above	90 (24)	28 (25)	62 (24)	
Having BF experience, n (%)				0.156
Yes	147 (39)	37 (33)	110 (42)	
No	227 (61)	74 (67)	153 (58)	
Exposure to BF education, n (%)				0.932
Yes	195 (52)	57 (51)	138 (52)	
No	179 (48)	54 (49)	125 (48)	
BF knowledge,Median (Q1-Q3)	11 (9- 14)	11 (9- 13.5)	11 (9- 14)	0.741
BF attitude, Mean ± SD	99.78 ± 13.67	98.83 ± 15.04	100.18 ± 13.06	0.41
Subjective norm, Median (Q1-Q3)	22 (18- 25)	22 (18- 25)	22 (18- 25)	0.772
Perceived control, Median (Q1-Q3)	34 (28- 43.75)	33(26.5- 43)	34 (28- 44)	0.227
PIM, Median (Q1-Q3)	17 (13- 21)	16 (12- 20)	17 (13- 21)	0.25
BF intention, Median (Q1-Q3)	10 (7.5- 14)	10 (7.25- 14)	10 (8- 14.25)	0.413
Delivery mode, n (%)				0.08
Vaginal delivery	148 (40)	52 (47)	96 (37)	
Caesarean section	226 (60)	59 (53)	167 (63)	
Maternity leave (days), Median (Q1-Q3)	180 (143- 180)	173(146.5–180)	180(143- 180)	0.857
EBF for 6 months, n (%)				0.958
Yes	46 (12)	13 (12)	33 (13)	
No	328 (88)	98 (88)	230 (87)	

*BMI* Body Mass Index, *BF* Breastfeeding, *PIM* Perception of insufficient milk, *EBF* Exclusive breastfeeding

### Univariable comparison of EBF and non-EBF groups

In the training set, participants were categorized into an EBF group and a non-EBF group based on the 6-month outcome. Univariable between-group comparisons are summarized in Table 2. Statistically significant differences were observed for maternal education, BF knowledge, BF attitude, subjective norm, perceived control, and BF intention (all *P* < 0.05). The variable “exposure to BF education” approached statistical significance in univariable testing (*P* = 0.053).

**Table 2:** Baseline characteristics between EBF and non-EBF in training set (*n* = 263)

Variables	Total (*n* = 263)	EBF (*n* = 33)	non-EBF (*n* = 230)	*p*
Age, Median (Q1-Q3)	30 (28- 33)	31 (29- 33)	30 (27- 33)	0.398
Pre-pregnancy BMI, Median (Q1-Q3)	22.95(20.69- 25.53)	22.77(20.69- 23.88)	23.04 (20.69- 25.7)	0.292
Quantity of child, n (%)				0.892
1	131 (50)	18 (55)	113 (49)	
2	121 (46)	14 (42)	107 (47)	
≥ 3	11 (4)	1 (3)	10 (4)	
Residence, n (%)				0.225
Urban	179 (68)	26 (79)	153 (67)	
Rural	84 (32)	7 (21)	77 (33)	
Average monthly household income (RMB), n (%)				0.758
< 3000	2 (1)	0 (0)	2 (1)	
3000–6000	30 (11)	3 (9)	27 (12)	
6000–10000	78 (30)	8 (24)	70 (30)	
> 10,000	153 (58)	22 (67)	131 (57)	
Education, n (%)				0.038
Junior college or below	201 (76)	20 (61)	181 (79)	
Bachelor degree or above	62 (24)	13 (39)	49 (21)	
Having BF experience, n (%)				0.792
Yes	110 (42)	15 (45)	95 (41)	
No	153 (58)	18 (55)	135 (59)	
Exposure to BF education, n (%)				0.053
Yes	138 (52)	23 (70)	115 (50)	
No	125 (48)	10 (30)	115 (50)	
BF knowledge, Median (Q1-Q3)	11 (9- 14)	14 (11- 16)	10.5 (8- 13)	< 0.001
BF attitude, Mean ± SD	100.18 ± 13.06	108.09 ± 13.41	99.05 ± 12.63	< 0.001
Subjective norm, Median (Q1-Q3)	22 (18- 25)	25 (24- 25)	21 (17- 25)	< 0.001
Perceived control, Median (Q1-Q3)	34 (28- 44)	44 (41- 47)	33 (28- 42)	< 0.001
PIM, Median (Q1-Q3)	17 (13- 21)	19 (14- 22)	17 (13- 20)	0.169
BF intention, Median (Q1-Q3)	10 (8- 14.25)	16 (13- 16)	9.5 (7.5- 13)	< 0.001
Delivery mode, n (%)				0.574
Vaginal delivery	96 (37)	14 (42)	82 (36)	
Caesarean section	167 (63)	19 (58)	148 (64)	
Maternity leave (days), Median (Q1-Q3)	180 (143- 180)	173 (158- 180)	180 (136.25- 180)	0.794

*BMI* Body Mass Index, *BF* Breastfeeding, *PIM* Perception of insufficient milk, *EBF* Exclusive breastfeeding

### LASSO-based feature selection

**Fig. 1:**
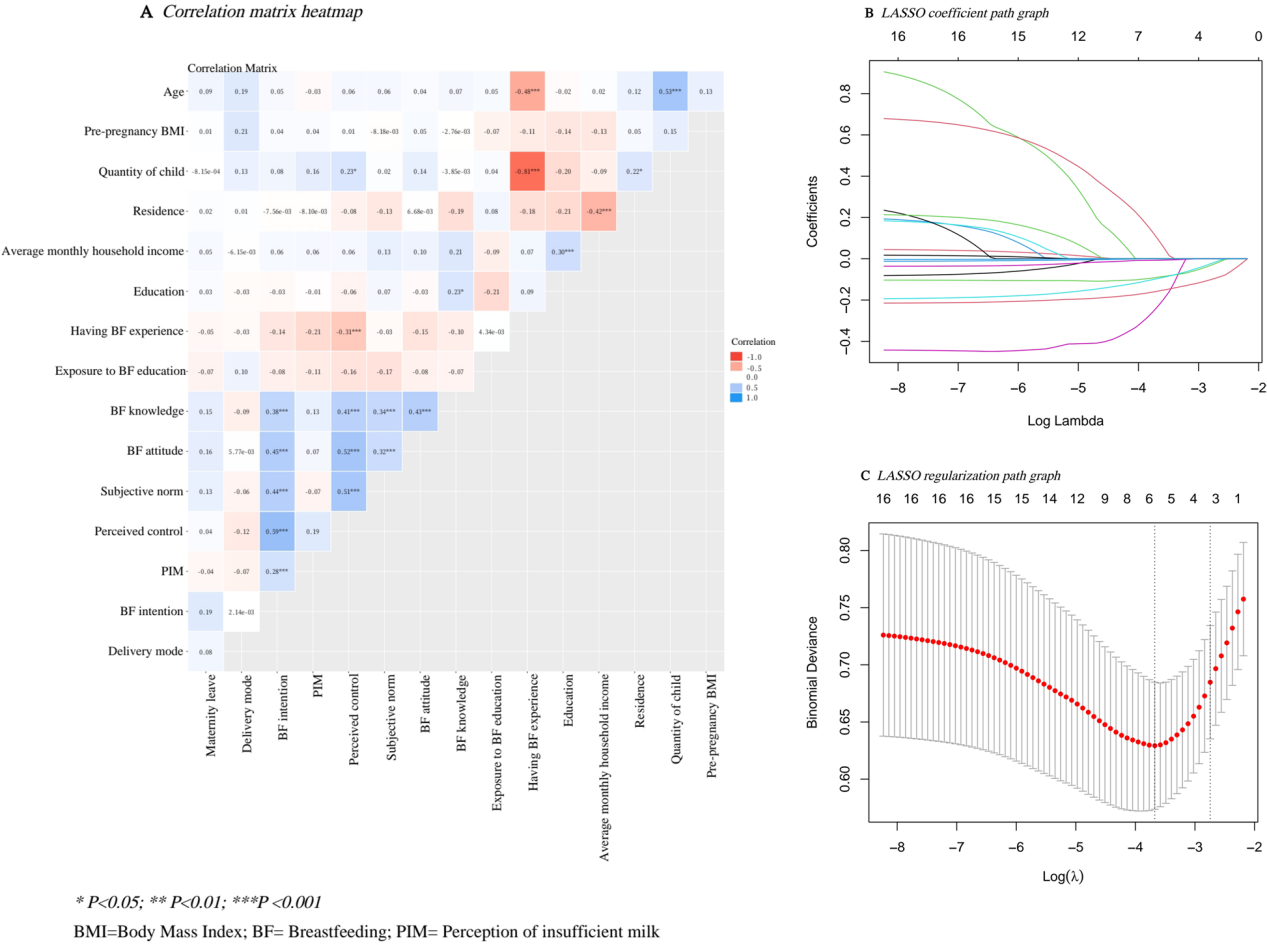
**A** Correlation matrix heatmap. **B** LASSO coefficient path graph. **C** LASSO regularization path graph

To assess potential correlations among candidate variables, a correlation matrix heatmap was generated (Fig. 1A). LASSO regression introduces an L1 penalty into the regression framework, which shrinks regression coefficients and enables data-driven feature selection (Fig. 1B). This approach may help alleviate potential multicollinearity among candidate variables and improve model generalizability and computational efficiency. The optimal lambda parameter was determined as the value that minimized cross-validated error (Fig. 1C). The final LASSO model identified maternal education, BF knowledge, BF attitude, subjective norm, perceived control, BF intention, and exposure to BF education as key predictors of 6-month EBF. To further assess statistical multicollinearity among these selected predictors, VIFs and tolerance values were examined. All the selected predictors demonstrated VIFs below 4 and tolerance values above 0.20, indicating no evidence of problematic multicollinearity (Table 3).

**Table 3:** Assessment of multicollinearity among selected predictors

	Unstandardized Coefficients		Standardized Coefficients	t	*p*	Collinearity Statistics	
	B	Standard Error	Beta			Tolerance	VIF
Constant	1.530	0.194		7.865	< 0.001		
Maternal education	−0.086	0.048	−0.110	−1.801	0.073	0.862	1.160
Exposure to BF education	0.037	0.039	0.056	0.940	0.348	0.916	1.092
BF knowledge	−0.010	0.007	−0.100	−1.450	0.148	0.679	1.473
BF attitude	−0.001	0.002	−0.031	−0.442	0.659	0.648	1.544
Subjective norm	−0.008	0.006	−0.099	−1.437	0.152	0.685	1.460
Perceived control	−0.002	0.003	−0.052	−0.641	0.522	0.491	2.038
BF intention	−0.017	0.006	−0.212	−2.881	0.004	0.597	1.675

*BF* Breastfeeding, *VIF* Variance Inflation Factor

### Model selection and performance evaluation

**Fig. 2:**
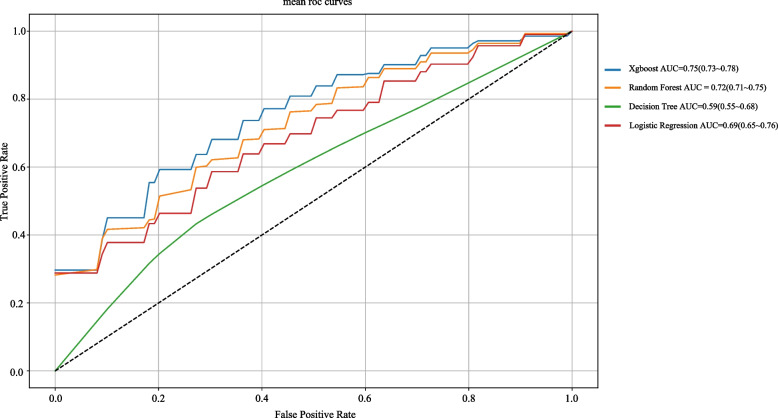
Model performance comparison for four algorithms

Using the LASSO-selected predictors, four models (XGBoost, decision tree, random forest, and logistic regression) were trained in the training set. Ten-fold cross-validation (repeated three times) was used to compare model performance within the training set. XGBoost achieved the highest mean AUC of 0.75 (95% CI: 0.73–0.78) and was identified as the best-performing model. Performance metrics for the other models, including random forest, decision tree, and logistic regression, are presented in Fig. 2.

### Performance of the XGBoost machine learning model

**Fig. 3:**
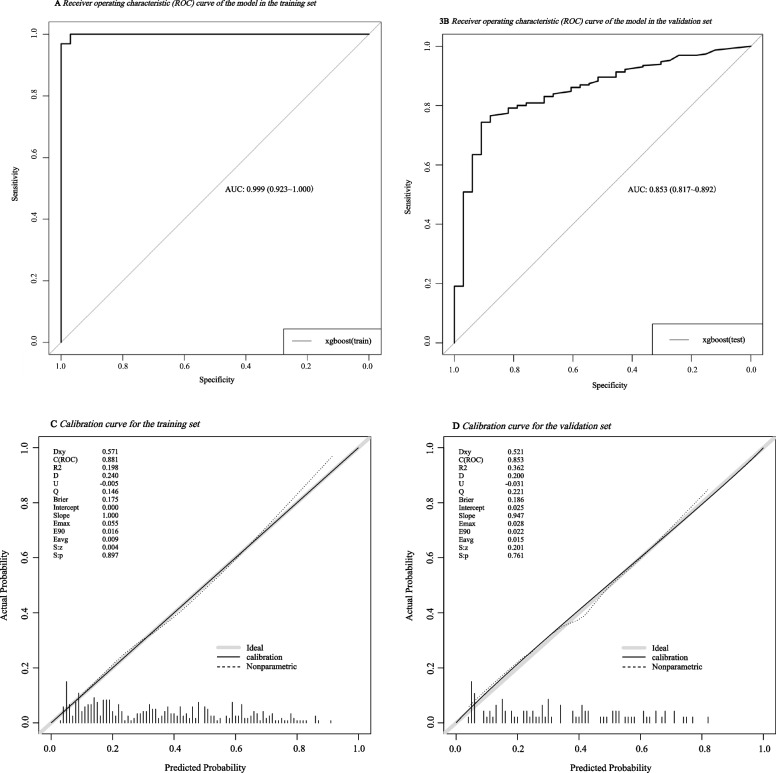
**A** Receiving operating characteristics (ROC) curve of the model in the training set. **B** Receiving operating characteristics (ROC) curve of the model in the validation set. **C** Calibration curve for the training set. **D** Calibration curve for the validation set

Building upon the results of model comparison, the XGBoost model was retrained on the full training set. In the training set, the model achieved an AUC of 0.999 (95% CI: 0.923–1.000) (Fig. 3A), sensitivity of 1.000, specificity of 0.939, precision of 0.991 and an F1-score of 0.995 (Table 4). In the validation set, the model yielded an AUC of 0.853 (95% CI: 0.817–0.892) (Fig. 3B), sensitivity of 0.935, specificity of 0.758, precision of 0.625 and an F1-score of 0.749 (Table 4). To further evaluate the reliability of model predictions in the context of potential overfitting, calibration analyses were conducted. The calibration curves demonstrated reasonable agreement between predicted and observed probabilities in both the training and validation sets (Fig. 3C and D). These findings indicate that, despite the observed drop in performance metrics, the XGBoost model achieved acceptable discrimination and reasonable calibration within the scope of internal validation.

**Table 4:** Performance of the XGBoost model in the training and validation sets

	**Train set**	**Test set**
AUC	0.999	0.853
Sensitivity	1	0.935
Specificity	0.939	0.758
Precision	0.991	0.625
F1 score	0.995	0.749

*AUC* Area under the receiver operating characteristic curve

### XGBoost machine learning model interpretation

**Fig. 4:**
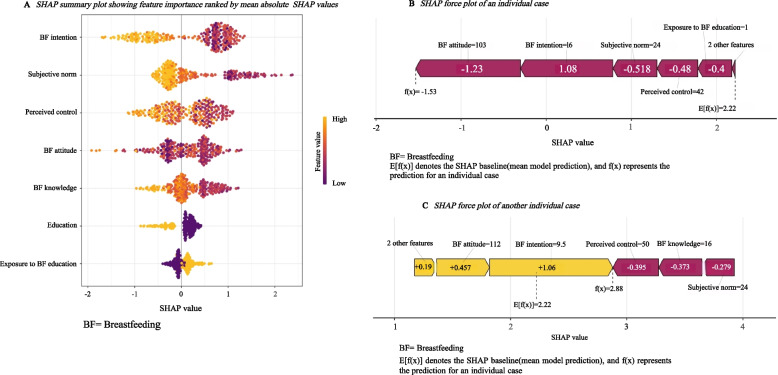
**A** SHAP summary plot showing feature importance ranked by mean absolute SHAP values. **B** SHAP force plot of an individual case. **C** SHAP force plot of another individual case

The SHAP summary plot illustrates the relative contribution of each predictor to the XGBoost model (Fig. 4A). Based on SHAP analysis, the variables were ordered by importance from highest to lowest as follows: BF intention, subjective norm, perceived control, BF attitude, BF knowledge, maternal education, and exposure to BF education. In the SHAP summary plot, yellow indicates higher feature values. Higher values (yellow) of BF intention, subjective norm, perceived control, BF attitude, BF knowledge, and maternal education were consistently associated with a higher likelihood of maintaining EBF during the first 6 months postpartum. In contrast, exposure to BF education is reverse-scored, with higher values (yellow) indicating no exposure, which increases the likelihood of discontinuing EBF.

To provide case-level interpretation, SHAP force plots were generated for 2 participants. In these plots, red bars represent features that support continued EBF (left shift), whereas yellow bars indicate features that increase the predicted probability of EBF discontinuation (right shift). The bar length denotes the strength of contribution, and the baseline value E[f(x)] corresponds to the mean model output. For a participant classified as maintaining 6-month EBF, the model predicted f(x) = –1.53, below the baseline, indicating continued EBF (Fig. 4B). Conversely, for a participant classified as non-EBF within 6 months, the model yielded f(x) = 2.88, above the baseline, indicating EBF discontinuation (Fig. 4C).

## Discussion

To address barriers to sustaining EBF within the Chinese context, we applied an explainable ML framework to identify determinants of 6-month EBF from multiple perspectives. Building on a data-driven modeling strategy involving preliminary feature selection and algorithm comparison, XGBoost was selected as the final predictive model for further interpretation. Beyond establishing predictive performance, the present analysis emphasized model interpretability, aiming to understand how multiple factors were weighted in relation to sustained EBF within the study cohort. To this end, SHAP analysis was used to examine and rank the relative contribution of individual predictors, highlighting BF intention, subjective norm, perceived control, BF attitude, BF knowledge, maternal education, and exposure to BF education as influential factors identified within the modeling framework.

Consistent with our findings, studies from Singapore and Changsha, China, identified maternal intention as a key factor in sustaining EBF during the first 6 months postpartum [26, 27]. According to the TPB, behavioral intention reflects an individual’s readiness and planned effort to perform a behavior, serving as the immediate driver of action [18]. In this context, mothers with stronger BF intentions are more likely to maintain EBF during the first 6 months. However, our results indicate that relatively few mothers expressed a firm intention to exclusively breastfeed for 6 months. This gap in intention may partly contribute to the challenges in achieving 6-month EBF within the study setting. In comparable settings with similar socioeconomic and healthcare characteristics, future research could further explore approaches to support the development of maternal BF intention as a potential direction for promoting sustained EBF.

Our findings also indicate that maternal BF attitude and subjective norm are key determinants of sustained EBF. BF attitude reflects a mother’s mindset or emotional orientation toward BF. It includes perceptions of both benefits and drawbacks. Mothers who hold more positive attitudes tend to be more motivated and persistent, making sustained EBF more likely. A previous study supported that women with favorable BF attitudes were more likely to maintain EBF for the recommended duration [28]. Another study also reported that higher BF attitude scores predicted greater adherence to EBF [29]. In this study, subjective norm denotes the perceived expectations from family, peers, and healthcare professionals regarding whether a mother should exclusively breastfeed. Higher subjective norm scores, indicating stronger social support for EBF, have been linked to longer EBF duration [30–32]. As an external social influence, subjective norm provides approval and reinforcement that reduce maternal uncertainty and strengthen persistence in EBF. These findings suggest that maternal attitude reinforces internal motivation, while subjective norm exerts external social support, highlighting their combined importance in improving the uptake and maintenance of EBF. In the setting studied, these insights may help inform the development of BF support approaches that nurture positive maternal beliefs and foster supportive social environments. They also provide a basis for future research examining whether similar patterns are observed in broader populations.

Perceived control emerged as another determinant of 6-month EBF. Our findings are supported by prior studies reporting that perceived control significantly predicts sustained EBF [32–34]. Perceived control reflects a mother’s confidence in her ability to maintain EBF despite potential barriers [35]. In the TPB, this construct was derived from Bandura’s concept of self-efficacy and is regarded as conceptually equivalent [36]. A strong sense of efficacy is essential for initiating behavioral change, whereas feelings of helplessness can reduce efforts to maintain positive behaviors [37]. Mothers with low perceived control may therefore be less motivated to continue EBF, while those who feel capable are more likely to take consistent action. These insights reinforce the theoretical relevance of perceived control within the TPB framework and highlight potential directions for further evaluation. Strengthening maternal perceived control, for example by building confidence through supportive counseling and improved access to resources, may represent a potentially relevant target for future research on EBF support strategies. However, its efficacy would need to be confirmed in external settings.

In addition, this study found that both BF knowledge and exposure to BF education contributed to the prediction of 6-month EBF. Mothers with more BF knowledge had greater odds of maintaining EBF to the recommended duration [29, 38]. BF knowledge represents a mother’s understanding of its benefits and skills. Lower levels of BF knowledge increase the likelihood of inappropriate feeding practices, such as the premature introduction of water or solids, which may lead to early termination of EBF [39]. Exposure to BF education has also been associated with improved EBF outcomes [40, 41]. Mothers may access such education through multiple channels, including antenatal classes, guidance from healthcare providers, and other organized educational programs. Although exposure to BF education did not reach statistical significance in univariable testing, it was retained in the final XGBoost model. BF knowledge captures actual understanding, whereas exposure to BF education reflects access to learning opportunities. These 2 variables may provide complementary information for predicting sustained EBF. Consistent with this study, prior studies also reported that women with higher education tended to maintain EBF for a longer duration [26, 42]. Within the study setting, this observation suggests that women with lower educational levels may benefit from additional attention in future BF support efforts.

### Strengths and limitations

This study has certain limitations that should be considered. The sample size was modest, and model performance was evaluated only through internal validation. Larger, multicenter studies are needed to conduct external validation and enhance generalizability. In addition, the study was conducted solely in Xuzhou, Jiangsu Province. Caution is warranted when applying these findings to populations with different cultural, socioeconomic, or healthcare contexts. Nevertheless, the study also has notable strengths. It is among the few prospective cohort studies in China that followed mothers for the entire 6-month postpartum period. This design minimized recall bias and provided more reliable estimates of EBF duration than cross-sectional studies. Integrating XGBoost for modeling with SHAP for interpretation yielded acceptable predictive performance and enhanced interpretability, thereby supporting the identification of key determinants of EBF. Furthermore, by considering demographic and socioeconomic, biomedical and clinical, belief and cognitive, and behavioral intention factors, the study provided a more comprehensive understanding of determinants of EBF in the Chinese context.

## Conclusions and future directions

This study applied a SHAP-interpretable XGBoost model, selected as the best-performing approach among 4 algorithms, to identify factors influencing the likelihood of sustaining 6-month EBF among mothers in Jiangsu Province, China. The analysis identified BF intention, subjective norm, perceived control, BF attitude, BF knowledge, maternal education, and exposure to BF education as key predictors of sustained EBF. These findings underscore the multifactorial nature of EBF behaviors and highlight the potential value of an interpretable, data-driven approach for examining factors related to sustained EBF within a specific local context.

Building on these insights, the findings generate preliminary, context-specific evidence that may help inform future research directions within this study setting. In this context, several integrated domains warrant further investigation. One potential area is the routine assessment of maternal BF intention during antenatal and postnatal care, together with individualized counseling to strengthen commitment. Future studies could also examine whether educational strategies that go beyond information provision by addressing misconceptions and fostering positive attitudes are associated with sustained EBF. In addition, further investigation is needed to determine whether approaches aimed at enhancing perceived behavioral control, such as skill training, and access to lactation support, are effective in addressing common BF barriers. The influence of partners, grandparents, and wider community networks on supportive subjective norms also merits closer examination. Moreover, future research may assess whether expanding structured BF education across healthcare, workplace, and community settings, including digital platforms, as well as tailoring support for mothers with lower educational attainment, can help reduce disparities in EBF. Consistent with the exploratory nature of the present analysis, further studies in more diverse populations, together with external validation, are required before considering applicability beyond this study context.

## Data Availability

The datasets used and analysed during the current study are available from the corresponding author on reasonable request.

## Abbreviations

AUC: Area under the receiver operating characteristic curve
BF: Breastfeeding
BMI: Body mass index
EBF: Exclusive breastfeeding
LASSO: Least absolute shrinkage and selection operator
ML: Machine learning
PIM: Perception of insufficient milk
SHAP: SHapley Additive exPlanations
TPB: Theory of planned behavior
VIF: Variance inflation factor
XGBoost: Extreme gradient boosting
